# How I treat HER2-low advanced breast cancer

**DOI:** 10.1016/j.breast.2023.01.005

**Published:** 2023-01-12

**Authors:** Ilana Schlam, Sara M. Tolaney, Paolo Tarantino

**Affiliations:** a Department of Hematology and Oncology, Tufts Medical Center, Boston, MA, USA; b Department of Medical Oncology, Dana-Farber Cancer Institute, Boston, MA, USA; c Division of New Drugs and Early Drug Development, European Institute of Oncology IRCCS, University of Milan, Milan, Italy

**Keywords:** Breast cancer, HER2-Low, Trastuzumab-deruxtecan, HER2

## Abstract

**Introduction:**

Targeting low levels of human receptor epidermal growth factor 2 (HER2) expression has reshaped the treatment paradigm for half of the patients with advanced breast cancer. HER2-low is currently defined as a HER2 immunohistochemical expression of 1+ or 2+ without amplification by in-situ hybridization. Until recently, HER2-targeted agents were ineffective in treating patients with HER2-low disease.

**Areas covered:**

In this narrative review, we summarize the current management of HER2-low breast cancer. We highlight the findings of the DESTINY-Breast 04 phase 3 trial, which confirmed the efficacy of trastuzumab-deruxtecan (T-DXd) for the treatment of patients with advanced, pretreated HER2-low breast cancer. We also discuss how to implement this new treatment option in treatment algorithms of hormone receptor (HR)-positive and triple-negative tumors, as well as how to optimally manage selected toxicities of T-DXd.

**Expert opinion:**

T-DXd is currently the standard of care for patients with advanced, pretreated, HER2-low breast cancer. Based on the design of the DESTINY-Breast04 trial, the current optimal place in treatment algorithms is after the first line of chemotherapy, both in HR-positive and triple-negative breast cancer. Up to 10–15% of the patients receiving T-DXd are expected to develop interstitial lung disease, which in 1–2% of the cases can be fatal. Adequate monitoring and prompt management are required to minimize the impact of ILD and to safely implement T-DXd in clinical practice.

## Highlights

• HER2-low is defined as a HER2 immunohistochemical expression of 1+ or 2+ without amplification by in-situ hybridization.
• HER2-low is not a distinct breast cancer subtype, but rather a target for potent, novel HER2-directed agents.
• Trastuzumab-deruxtecan (TDX-d) is approved for the treatment of pretreated, advanced HER2-low breast cancer.
• Education about the risk of interstitial lung disease and cardiac toxicity is needed prior to the initiation of TDX-d.

## 1 Introduction

Two point three million women are diagnosed yearly with breast cancer, and 685,000 breast cancer-related deaths occur annually worldwide [1]. By the end of 2020, there were 7.8 million women alive who were diagnosed with breast cancer between 2015 and 2020, making it the most prevalent cancer [1]. Three breast cancer subtypes have been defined based on widely available biomarkers: estrogen receptor (ER), progesterone receptor (PR), and/or human epidermal growth factor receptor 2 (HER2) [2]. The expression of these receptors by tumor cells plays a key role in cancer treatment and prognosis of patients with breast cancer [2]. ER and/or PR expressing tumors account for 70% of all breast cancers and are known as hormone receptor (HR) positive breast cancers [2]. Around 15% of breast cancers overexpress HER2, from which half also express ER and/or PR [2]. The remaining 15% do not overexpress HR or HER2; this subtype is known as the triple-negative breast cancer (TNBC) [2].

Assessing HER2 expression in patients with early and advanced breast cancer is critical; HER2-targeted therapies have dramatically improved the prognosis of patients with HER2-positive disease [3,4]. HER2 expression can be assessed by immunohistochemistry (IHC) and by in-situ hybridization (ISH). Based on current guidelines, HER2-positivity is defined as tumors with IHC 3+ for HER2 or IHC 2+ with a HER2/chromosome enumeration probe 17 (CEP17) ratio ≥2 and/or a HER2 copy number ≥6 signals/cell by ISH [5]. Even though only around 15% of tumors meet these criteria, around 30–60% of the tumors traditionally defined as “HER2-negative” show low levels of HER2 expression, in the absence of gene amplification [[6], [7], [8]]. This subset of tumors is now known as HER2-low breast cancer and is defined as IHC 1–2+ without amplification with ISH [9]. This large subset of patients represents an attractive area of opportunity for HER2-targeted therapies.

The first HER2-targeted treatment to be extensively studied for the HER2-low disease was trastuzumab. NSABP B-47 was a phase 3 trial in which 3270 women with high-risk, HER2-low breast cancer were randomized to chemotherapy with or without trastuzumab between 2011 and 2015 [10]. At a median follow-up of 46 months, there was no difference in invasive disease-free survival (iDFS) between the groups, irrespective of hormone receptor status, HER2 IHC expression, or lymph node involvement [10]. Similarly, a phase 2 study showed little benefit with pertuzumab [11] for the treatment of patients with HER2-low breast cancer, and a retrospective analysis looking at trastuzumab-emtansine (T-DM1) in HER2-non-amplified tumors found little activity. Based on these findings, the use of HER2-targeted agents was limited to patients with HER2-positive tumors. However, with the development of more potent HER2-directed therapies, such as novel antibody-drug conjugates (ADC), the question about the use of HER2-targeted agents for HER2-low breast cancer re-emerged and gained momentum, particularly after the publication of the results of DESTINY-Breast04 [12]. This study confirmed the efficacy of the ADC trastuzumab-deruxtecan (T-DXd) for the treatment of patients with advanced, pretreated HER2-low breast cancer; this trial is discussed in detail in this manuscript.

T-DXd is an ADC composed of a HER2-targeted monoclonal antibody, a cleavable linker, and the cytotoxic payload is deruxtecan, a potent topoisomerase inhibitor I [13]. T-DXd has a high drug-to-antibody ratio of 8:1 [13]. Once deruxtecan is released in the HER2-positive cancer cell, it can diffuse out of the cell and can have a cytotoxic effect on surrounding HER2-negative tumor cells and on the tumor microenvironment, a characteristic known as “bystander effect” [13].

The use of ADCs in HER2-low breast cancer has reshaped the treatment paradigm of advanced breast cancer. However, multiple questions remain to determine how to incorporate the treatment of HER2-low breast cancer in the rapidly evolving treatment landscape of the traditionally known hormone receptor-positive and triple-negative breast cancers. In this narrative review, we discuss the current treatment algorithm for HER2-low breast cancer and frequent clinical challenges.

## 2 Epidemiology, biology, and prognosis of HER2-low breast cancer

The rate of HER2-low breast cancer is around 40–65% in HR-positive tumors, and 23–40% of the HR-negative tumors express low levels of HER2 expression [6,7,14]. Notably, the rate of HER2-low expression appears to increase with ER expression [14]. It has been well established that HER2 expression is dynamic and that it can change as the disease progresses with up to 40% discordance between primary and metastatic tumors; HER2-low expression appears to be enriched in the advanced setting [15,16]. These findings underscore that, when feasible and safe, it is useful to obtain repeat biopsies at the time of progression of disease, as these can impact treatment options.

An important clinical question is if the large subset of tumors with HER2-low expression represents a distinct biological entity. This question could be addressed in several ways: one is to determine if HER2-low tumors are biologically different from HER2-zero tumors, while another possibility is to determine if this biomarker confers patients with a unique prognosis.

A study collected clinicopathologic and PAM50 intrinsic subtype data from 3689 patients with HER2-negative breast cancer; 71% of samples were from primary breast tumors. The investigators found that HR-positive tumors had higher rates of HER2-low disease [6]. HER2-low tumors had larger primary tumor sizes (p = 0.007) and more nodal involvement (p = 0.010); there were no differences in Ki67 scores or in the percentage of tumor-infiltrating lymphocytes (TILs) between HER2-zero and HER2-low expression [6]. PAM50 data were available from 1576 patients: most of the HR-positive/HER2-low tumors were more frequently Luminal A or B (79.6%), while HR-negative/HER2-low tumors were most frequently Basal-like (84.7%) or HER2-enriched (8.5%); these findings were confirmed by Agostinetto et al. and are consistent with prior studies assessing HR-positive and TNBC [6,16,17]. Gene expression analysis showed findings consistent with the mentioned intrinsic subtypes [6].

**Fig. 1:**
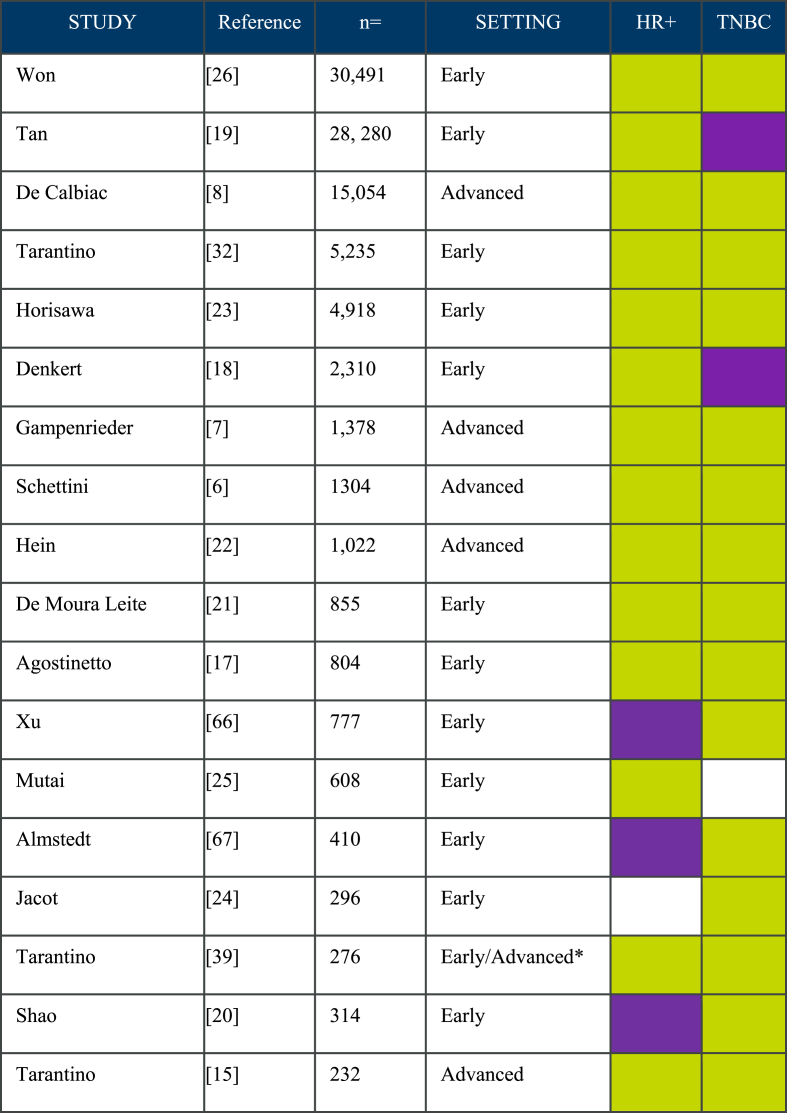
Summary of selected studies conducted to determine the prognostic significance of HER2-low disease [[21], [22]]. **Abbreviations:** HER2: human epidermal growth factor receptor 2, HR+: hormone receptor-positive, n= sample size, TNBC: triple-negative breast cancerThe studies that showed no difference in overall survival are marked in green , and those that showed a statistically significant difference in overall survival in purple (White = not evaluated).*Inflammatory breast cancer only

Multiple studies have been conducted to assess the prognostic implications of HER2-low expression in early and advanced breast cancer (Summarized in Fig. 1). Three of these studies, which included a total of 47,145 patients, have reported differences in outcomes for patients with early HER2-low disease [8,18,19]. An analysis of 28,280 patients with early breast cancer by Tan et al., showed that the outcomes for patients with HER2-low breast cancer were better than for those with HER2-zero disease, including recurrence-free interval (Hazard ratio 0.88, 95% CI 0.82–0.93, p = 0.001) and OS (Hazard ratio 0.82, 95% CI 0.76–0.89, p = 0.001); these differences were consistent irrespective of HR-expression [19]. A cohort study assessing samples from 15,054 patients with HER2-negative metastatic breast cancer treated in France between 2008 and 2016 revealed that 31% of the tumors could be reclassified as HER2-low [8]. With a median follow-up of 50 months, the median OS was 38 months in the HER2-low group vs 33.9 in the HER2-zero (p < 0.001), there was no difference in PFS [4]. In a pooled analysis of 2310 patients with breast cancer that received neoadjuvant therapy, Denkert et al. showed patients with HER2-low tumors had longer overall survival (OS) rates, relative to those with HER2-zero disease (3-year OS rate was 85.8% vs 91.6%, p = 0.0016) (18). Patients with HR-positive/HER2-low tumors had lower rates of pathologic complete response (pCR), relative to HR-positive/HER2-zero tumors; this difference was not seen in HR-negative/HER2-low tumors [18]. In contrast, Saho et al. studied a cohort of 314 patients, in which patients with, HR-positive/HER2-low tumors had higher rates of pCR, relative to HR-positive/HER2-zero tumors [20].

Conversely, 11 studies that included a total of 47, 419 patients were designed to answer the question about the prognostic implication of HER2-low disease and failed to show differences in outcomes, irrespective of the HR status [6,7,14,15,17,[23], [24], [25], [26], [27], [28]]. In conclusion, based on the small differences reported in the positive studies and the large number of studies that haven't shown differences in prognosis, HER2-low does not appear to be a biological distinct breast cancer subtype; but rather, low levels of HER2-expression could be used as a predictive marker of response to therapies, as seen on DESTINY-Breast04 [12].

## 3 A new standard for HER2-low metastatic breast cancer: DESTINY-Breast04

DESTINY-Breast04 was a phase 3 trial in which 557 patients with pretreated (endocrine treatment if HR-positive, plus 1–2 prior lines of chemotherapy), advanced HER2-low breast cancer were randomized 2:1 to receive T-DXd or chemotherapy of physician's choice [12]. The primary endpoint of this study was progression-free survival (PFS) in the HR-positive cohort [12].

The study population included 494 (88.7%) patients with HR-positive disease and 63 (11.3%) with HR-negative disease, 58% of patients had HER2 IHC 1+ and 42% IHC 2+; the liver was the most common site of metastatic disease, followed by lungs [12]. In terms of prior therapies, all patients had received at least one line of chemotherapy; 99.7% of the HR-positive cohort received prior endocrine therapy, while 70% of that cohort received prior CDK4/6 inhibitor [12]. In the intention to treat population, the median PFS was 9.9 months in the T-DXd arm and 5.1 months in the chemotherapy arm (hazard ratio 0.50, P < 0.001), and OS was 23.4 months and 16.8 months, respectively (hazard ratio 0.64, P = 0.001). In the HR-positive cohort, the median PFS was 10.1 months vs 5.4 months, favoring T-DXd (Hazard ratio 0.51, p < 0.001), and the median OS was 23.9 months vs 17.5 months (Hazard ratio 0.64, p = 0.003) [12]. In the HR-negative population, the median PFS was 8.5 months vs 2.9 months (Hazard ratio 0.46, 95% CI 0.24–0.89), and the median OS was 18.2 months vs 8.3 months (Hazard ratio 0.48, 95% CI 0.24–0.95) [12]. The findings were seen irrespective of HER2 expression (IHC 1+ or 2+).

More patients in the T-DXd arm experienced adverse events that led to treatment discontinuation (16.2% vs 8.1%) compared to the control arm [12]. In terms of toxicities, alopecia, nausea, vomiting, constipation, diarrhea, anemia, and thrombocytopenia were more frequent in the T-DXd arm; while neutropenia was more frequent in the chemotherapy arm [12]. Drug-related interstitial lung disease (ILD) occurred in 45 (12.1%) of the patients who received T-DXd, including 13 with grade 1, 24 with grade 2, 5 with grade 3, and 3 (0.8%) with a grade 5 ILD. A total of 17 (4.6%) of the patients on the T-DXd arm experienced decreases in the left ventricle ejection fraction. The findings from DESTINY-Breast04 led to the approval of T-DXd by the United States Food and Drug Administration (US FDA) in August of 2022 for patients with pretreated HER2-low metastatic breast cancer who have received at least one line of prior chemotherapy.

## 4 Adverse events of interest: gastrointestinal, pulmonary, and cardiac toxicities

Gastrointestinal toxicities are frequent in patients treated with T-DXd. Nausea has been reported in 72.8–77.5% of patients (including 4.6–7.6% grade 3), vomiting in 34–45% (1.3–4.3% grade 3), constipation in 21–35.9% (0.5% grade 3), diarrhea 22.4–27.3% (0.4–1.1% grade 3), and abdominal pain in 16.8% (1.1% grade 3) [13,29,30]. T-DXd is moderately emetogenic; nausea and vomiting are dose-limiting toxicities that can affect significantly patient's quality of life. The current recommendations include starting at least with a two-drug anti-emetic regimen, for example, dexamethasone and ondansetron and considering a three-drug regimen upfront or escalation of treatment depending on the patient's symptoms.

ILD is of significant concern and should be taken into consideration when treating patients with T-DXd (Table 1 summarizes the rates of ILD in studies assessing the use of T-DXd for the treatment of patients with breast cancer). Based on animal models, T-DXd-induced ILD appears to be dose-dependent and related to the effect of the membrane soluble cytotoxic payload in the alveolar macrophages [31]. Deruxtecan may be causing direct alveolar inflammation, and this toxicity does not seem to be an on-target effect of the HER2-directed antibody [31].

**Table 1:** Summary of adverse events of interest in selected trastuzumab-deruxtecan trials.

Trial	Reference	Sample size	ILD		Cardiomyopathy	
			Any grade	Grade 3 or higher	Any grade	Grade 3 or higher
NCT02564900 (Phase 1)	[32]	115	20 (17.3%)	3 (2.6%)	0	0
DESTINY-Breast 01 (Phase 2)	[33]	184	28 (15.2%)	5 (2.7%)	5 (2.7%)	3 (1.6%)
DESTINY-Breast 02 (Phase 3)	[34]	608	42 (10.4%)	5 (1.2%)	18 (4.5%)	2 (0.5%)
DESTINY-Breast 03 (Phase 3)	[35]	524	39 (15.2%)	2 (0.8%)	6 (2.3%)	0
DESTINY-Breast 04 (Phase 3)	[12]	494	45 (12.5%)	8 (1.6%)	17 (4.6%)	5 (1.5%)

Abbreviations: interstitial lung disease.

ILD was first noted in the initial studies of T-DXd for HER2-positive breast cancer. In a phase 1 study (NCT02564900) assessing the role of T-DXd for patients with HER2-positive metastatic solid tumors, 115 patients with pretreated breast cancer were included and 17.3% (n = 20) cases of ILD/pneumonitis/organizing pneumonia were reported, including one grade 3 event and two treatment-related deaths due to pneumonitis [32]. DESTINY-Breast 01 was a single arm, phase 2 study in which 184 patients with heavily pretreated (median number of prior lines of treatment was 6, range 2–27) HER2-positive advanced breast cancer were treated with T-DXd [33]. This study showed a median duration of response of 14.8 months (95% CI 13.8–16.9) and median PFS of 16.4 months and led to the approval of T-DXd for patients with HER2-positive advanced breast cancer [33]. Notably, 15.2% (n = 28) of patients in this study developed ILD, including one grade 3 event and four treatment-related deaths due to pneumonitis [12,36]. DESTINY-Breast 02 was a phase 3 trial comparing T-DXd vs treatment of physician's choice in 608 patients with pretreated (median 2–3 prior lines of treatment and nearly all received prior trastuzumab emtansine) HER2-positive advanced breast cancer [34]. At a median follow up of 20 months, the median PFS was 17.8 months with T-DXd and 6.9 months with chemotherapy (HR 0.36, 95% CI 0.28-0.45, p < 0.00001) and OS of 39.2 vs 26.5 months, respectively (HR 0.66, 95% CI 0.50-0.86, p = 0.0021) [34]. A total of 10.4% of patients developed ILD [34]. DESTINY-Breast 03 was a phase 3 trial comparing T-DXd and the ADC trastuzumab emtansine (T-DM1) for 524 taxane/trastuzumab pretreated patients with metastatic HER2-positive breast cancer, at a median follow up of 28 months, the PFS was 28.8 months vs 6.8 months favoring T-DXd (HR 0.33, 95% CI 0.26-0.43, p < 0.000001), there was also an improvement in OS (41 vs 34 months, HR 0.64, 95% CI 0.47-0.87, p0037) [29,35]. Notably, the rate of ILD was 15.2% (n = 39), including two grade 2 events, and no treatment-related deaths related to ILD were reported. The absence of fatal ILD events in DESTINY-Breast03 suggests that patients with more heavily pretreated disease may be at higher risk for severe ILD, additionally, increased awareness on the risk of ILD could have reduced its severity. As mentioned before, in DESTINY-Breast 04, ILD occurred in 12.1% (45) of patients with HER2-low disease treated with T-DXd, including five grade 3 events, and three treatment-related deaths; the median number of prior therapies in this study was 3 (Range 1–9) [12]. These findings also suggested that more heavily pretreated patients may be at higher risk for high-grade ILD.

In terms of monitoring and management of ILD, patient and provider education are critical. Most cases of ILD occur within the first year of treatment [37]. New respiratory symptoms and suspicious pulmonary changes in restaging scans should raise concern as a high index of suspicion by healthcare providers is needed for a timely diagnosis and treatment of patients with ILD [38]. Patients with new respiratory symptoms should undergo a high-resolution CT scan; additional testing, including bronchoscopy and pulmonary function tests can be considered in case-by-case basis [39]. Asymptomatic patients are usually followed with scans every 6–12 weeks. For asymptomatic ILD (Grade 1) the recommendation is to hold T-DXd and to consider steroid use, and if the symptoms resolve, re-challenge could be considered [40]. In cases of symptomatic ILD (Grade 2 or greater), steroids are recommended with a slow taper [41]. Patients with HER2-low disease may have received prior therapies which are associated to pneumonitis, including immune checkpoint inhibitors, everolimus and CDK4/6 inhibitors. The mechanism of action of these toxicities appears to be different; however, it remains unclear if patients with prior treatment-related pneumonitis are at higher risk for T-DXd included ILD.

Given that T-DXd contains a HER2-directed monoclonal antibody, cardiomyopathy is another toxicity of interest. A transthoracic echocardiogram (TTE) is recommended prior to treatment initiation, and ejection fraction (EF) monitoring with TTE every three months is advised [40]. Table 1 summarizes the rates of cardiomyopathy in studies assessing the use of T-DXd for the treatment of patients with breast cancer. The rates of decreased EF are similar to the ones reported for trastuzumab trials [42]. Although the patient numbers are small, in DESTINY-Breast03, one patient treated with T-DM1 was found to have a decreased EF and six in the T-DXd arm; more information is needed to determine if there are differences in safety between the HER2-directed ADCs [29].

## 5 Expert opinion: incorporating HER2-low in the current clinical practice

The approval of T-DXd for the treatment of HER2-low advanced breast cancer reshaped the treatment paradigm for more than half of the patients with metastatic breast cancer; however, several questions remain. A recurrent clinical challenge is how to incorporate this drug in the treatment of advanced breast cancer. Given that HER2-low breast cancer does not appear to represent a distinct breast cancer subtype, commonly used biomarkers and treatment should be used to guide the initial treatment decisions. There is currently no role for HER2-directed therapies for patients with early breast cancer with HER2-low expression outside of clinical trial, irrespective of the HR-status.

**Fig. 2:**
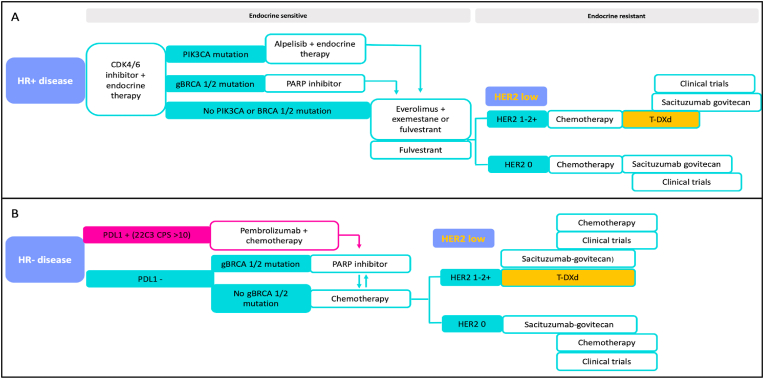
Proposed treatment algorithm for hormone receptor-positive (A) and hormone receptor-negative (B) HER2-low breast cancer. **Abbreviations**: CDK: cyclin dependent kinase, gBRCA 1/2: germline BRCA 1 or 2 pathogenic variant, HER2: human epidermal growth factor receptor 2, HR: hormone receptor, PARP: poly-ADP ribose polymerase, PDL1: program cell death ligand 1, T-DXd: trastuzumab deruxtecan

Patients with HR-positive/HER2-negative tumors that have endocrine-sensitive disease are treated with endocrine therapy, often in combination with novel agents, such as CDK4/6 [43,44] or PIK3 inhibitors [45]. Olaparib and talazoparib are PARP inhibitors approved for the treatment of patients with pathogenic or likely pathogenic germline BRCA 1 or 2 variants [46,47]. Once the tumor is considered endocrine-resistant, patients often receive one line of chemotherapy. Then those with HER2-zero tumors could be treated with the TROP2-targeted ADC sacituzumab-govitecan [48], while patients with HER2-low expression, could be treated with either T-DXd or sacituzumab-govitecan [12,48], followed by other lines of chemotherapy and consideration for clinical trials [12,49]. Fig. 2A shows our proposed treatment algorithm for HR-positive/HER2-low breast cancer. The optimal sequencing of ADCs in this setting has not been established and trials comparing these agents head-to-head are not available. However, based on the significant improvement in PFS and OS seen in DESTINY-Breast 04, as well as the less pretreated population included in DESTINY-Breast04 compared to TROPiCS02, T-DXd could be considered first. In DESTINY-Breast 04, patients with HR-positive/HER2-low disease did not receive endocrine therapy concurrently with T-DXd, and the efficacy of this combination is unclear, thus not currently advised in clinical practice. There are ongoing trials assessing the role of this combination for early and advanced HER2-low breast cancer (Trials are summarized in Table 2).

**Table 2:** Ongoing studies for HER2-low breast cancer (clinicaltrials.gov as of December 9, 2022).

Study/Phase	Design	n =	Outcome measures
Early disease			
NCT05165225 (Phase 2)	Single arm. Pyrotinib combined with neoadjuvant chemotherapy in early or locally advanced disease	46	Primary: pCR Secondary: grade of pCR, breast conservation rate, DFS, OS, biomarkers, incidence of grade 3 diarrhea
NCT04553770 (Phase 2)	Neoadjuvant T-DXd x 6 or T-DXd x 6 + anastrozole for early stage HR+/HER2 low disease	88	Primary: pCR Secondary: incidence of adverse events, changes in biomarkers, clinical objective response, biomarker analyses, quality of life questionnaire
Cornerstone001 - NCT05163223 (Phase 2)	Patients with residual disease after neoadjuvant therapy for HR-/HER2 low breast cancer will be randomized to the HER2-cancer vaccine AST-301(pNGVL3-hICD) + chemo -immunotherapy vs Placebo + chemo -immunotherapy	146	Primary: iDFS Secondary: immune response to AST-301(pNGVL3-hICD), change in memory T-cell populations, dRFS, treatment related toxicities
**Advanced disease**			
NCT05461768 (Phase 1)	BL-B07D1 (HER2-targeted ADC) for pretreated HER2-positive or low advanced breast cancer	26	Primary: dose-limiting toxicity, maximum tolerated dose, clinical study recommended dose Secondary: toxicities, pharmacokinetics, ORR, DCR, DOR, PFS
ISPY-P1.01 - NCT04602117 (Phase 1)	Basket trial assessing weekly paclitaxel and T-DXd for patients with solid tumors, including metastatic HER2-low breast cancer	27	Primary: toxicities, clinical benefit rate, ORR Secondary: PFS, DOR
NCT04147819 (Phase 1)	BAY2701439 Is a ‘targeted alpha therapy’ which uses an antibody to deliver a radioactive particle (thorium-227) to cancer cell. One of the four cohorts is for HER2-low advanced breast cancer	213	Primary: dose escalation, dose expansion Secondary: Pharmacokinetics of the antibody and radioactive particle
DESTINY-Breast 08 - NCT04556773 (Phase 1 b)	T-DXd + capecitabine or T-DXd + durvalumab + paclitaxel or T-DXd + capivasertib or T-DXd + anastrazole or T-DXd with fulvestrant. Each combination has a dose-finding and a dose-expansion part.	182	Primary: occurrence of adverse events in each group Secondary: ORR, PFS, DoR, OS, serum concentration of T-DXd and durvalumab, immunogenicity of T-DXd and durvalumab
NCT05018676 (Phase 2)	Single arm: ARX788 (HER2-targeted ADC) for pretreated HER2-low cancer	54	Primary: ORR Secondary: PFS, OS, DCR
NCT04742153 (Phase 2)	Single arm: MRG002 (HER2-targeted ADC) for pretreated HER2-low cancer	66	Primary: ORR Secondary: PFS, TTR, DoR, DCR, OS, toxicities, pharmacokinetics, immunogenicity
DESTINY-Breast 06 - NCT04494425 (Phase 3)	T-DXd vs treatment of investigators choice for HR+/HER2 low advanced breast cancer	850	Primary: PFS Secondary: OS, ORR, DoR, PFS2, tolerability, immunogenicity of T-DXd, patient reported outcomes
NCT04400695 (Phase 3)	RC48-ADC (HER2-targeted ADC) vs treatment of investigators choice for HER2 low advanced breast cancer	366	Primary: PFS Secondary: ORR, DOR, DCR, TTP, OS

Abbreviations: ADC: antibody-drug conjugate, DCR: disease control rate, DoR: duration of response, dRFS: distant recurrence-free survival, HER2: human epidermal growth factor receptor 2, HR+: hormone receptor-positive, iDFS: invasive disease-free survival, n = sample size, pCR: pathologic complete response, ORR: objective response rate, OS: overall survival, PFS: progression-free survival, T-DXd: trastuzumab deruxtecan, TTP: time to progression, TTR: time to response.

T-DXd was initially approved for patients with HER2-positive breast cancer who had received at least two prior lines of treatment based on DESTINY-Breast 01 [13], this drug was then approved in the second line setting based on DESTINY-Breast 03 [29] and there are ongoing studies to assess the efficacy of this agent in the first line setting (DESTINY-Breast 09, NCT04784715). In HER2-low disease, T-DXd is currently approved for patients who have received at least one prior line of chemotherapy and studies such as DESTINY-Breast 06 (NCT0449425) are assessing if this medication could move to the first line setting, once endocrine therapies are exhausted in HR-positive, HER2-low advanced breast cancer.

The targeted treatment options for patients with advanced TNBC have increased over the past few years. Program cell death ligand expression (PDL1) by the Dako 22C3 assay should be assessed at diagnosis; patients with a combined positive score ≥10 are candidates for first-line treatment with pembrolizumab in combination with chemotherapy [50]. Patients who are not candidates for immunotherapy are often treated with first-line chemotherapy. Olaparib and talazoparib are approved for patients with pathogenic or likely pathogenic germline BRCA 1 or 2 variants [46,47]. For patients with HER2-low expression, after at least one line of chemotherapy, T-DXd or sacituzumab govitecan should be considered; while those with HER2-zero tumors could be treated with sacituzumab govitecan followed by other lines of chemotherapy and consideration for clinical trials [12,51]. Fig. 2B illustrates our proposed treatment algorithm for HR-negative/HER2-low breast cancer.

The optimal treatment sequencing beyond first line chemo-immunotherapy for eligible patients, has not been established. There are currently two ADCs approved in this setting. Sacituzumab govitecan was approved based on the phase 3 ASCENT trial [52], which included 233 patients with TNBC, while T-DXd was approved based on the DESTINY-Breast 04 [12] in which only 63 patients had HR-negative, HER2-low disease. Despite the limited representation of these patients, T-DXd represents an attractive treatment option. One must consider prior (neo)adjuvant therapies and disease-free interval, toxicity profile, comorbidities as well as patient preference when selecting treatments. It remains unclear if there is cross-resistance, as they both have topoisomerase 1 payloads, and the optimal sequencing of these novel agents for patients with HER2-low disease is unknown. There are other ADCs in advanced phases of development which will likely increase the treatment armamentarium of advanced TNBC. An example is datopotamab deruxtecan, which shares the deruxtecan payload with T-DXd and the TROP2 target with Sacituzumab, this drug has shown promising results in early phase studies and phase 3 studies are ongoing [53].

HER2 testing represents another clinical challenge. This problem has gained significance as it is critical to determine if a tumor has zero or 1+ HER2 expression to guide the treatment of patients with HER2-low breast cancer [54]. The assay used for IHC testing is relevant, as there is discordance between the commercially available assays [55]. It was previously reported that there is only 73.2% concordance between the Dako HercepTest and Ventana 4B5 assay [55]. Notably, a more recent version of the HercepTest showed up to 98.2% concordance with the 4B5 assay in breast cancer samples; HercepTest was more sensitive than 4B5 [56]. Additional studies are required to determine if one assay is better than the other as a predictive marker for response to HER2-targeted agents. There is also a high rate of discordance among the pathologists analyzing HER2 IHC; a recent study revealed only 26% concordance in HER2-zero and 1 among 18 experienced pathologists [54]. These findings underscore the urgent need for novel quantitative HER2 assays. Several ongoing studies are assessing methods to quantify HER2 expression, including DNA, mRNA, and protein-based assays [[57], [58], [59]]. These are likely to optimize HER2 testing with the goal of providing targeted therapies for patients who are likely to derive a benefit while limiting exposure to treatment and toxicity to patients who are less likely to benefit.

There are other subsets of patients who could benefit from HER2-directed therapies, including patients with HER2-low breast cancer brain metastases (BCBM) and those with “ultralow” HER2 expression. T-DXd has shown central nervous system penetrance and efficacy for patients with HER2-positive BCBM in the DEBBRAH and TUXEDO-1 studies, in a retrospective (ORR was 73%) cohort of 17 patients, and in a subset analysis of the mentioned DESTINY-Breast 01 [[60], [61], [62], [63]]. DESTINY-Breast 12 (NCT04739761) is a phase 3 trial assessing the role of T-DXd in patients with and without HER2-positive BCBM. In terms of HER2-low BCBM, DAISY (NCT04132960) was a phase 2 clinical trial that included 10 patients with asymptomatic HER2-low BCBM (7 also expressed HR) the best objective response was 30%, clinical benefit ratio 50% and the median PFS was 4.1 months [64]. Additionally, a cohort of the DEBRAH trial (NCT04420598) is assessing the efficacy of T-DXd in this patient population. In terms of HER2 “ultralow”, it is well known that cancer cells express HER2, and it can only be detected and characterized by the currently available methods at a certain level of overexpression and/or amplification. Since T-DXd demonstrated efficacy in HER2-low tumors, the question of targeting the undetectable HER2 receptors was raised. The phase 2 DAISY trial included 3 arms: one of HER2-overexpressing tumors (n = 72), one of HER2-low expressing (n = 74), and one arm of HER2 IHC 0 tumors (n = 40) [65]. Best overall response was 69.1%, 33.3%, and 30.6%, and the median PFS was 11.1, 6.7 and 4.2 months, respectively [65]. These findings suggest that selected patients with HER2 ultralow disease could benefit from HER2-directed therapies, and improved testing methods will likely provide additional tools to improve patient selection for clinical trials [65].

ADCs are also being studied in the neoadjuvant setting. NeoSTAR was a phase 2 trial in which 50 patients with early TNBC were treated with four cycles of sacituzumab govitecan, the pathologic complete response rate was 30%, other arms of this study are currently enrolling patients (NCT04230109) [66]. The TALENT trial assessed the role of six to eight cycles of T-DXd with or without anastrozole in the neoadjuvant setting in 58 patients with HR-positive and HER2-low stage 2–3 breast cancer [67]. The objective response rate was 68% in the T-DXd alone arm and 58% in the T-DXd and anastrazole arm, long term and translational outcomes of this trial have not been reported. ADCs are currently not standard of care in the neoadjuvant setting, however the mentioned studies as well as several ongoing trials will continue to provide information to determine if these agents could be incorporated in the (neo)adjuvant to continue to improve patient outcomes.

In conclusion, HER2-directed treatment with T-DXd has reshaped the treatment paradigm of patients with HER2-low disease, irrespective of the HR-receptor status. Multiple questions remain unanswered and several trials are being conducted (summarized in Table 2) to answer some of the questions and expand the role of HER2-low as a target in other settings, such as early, high-risk disease.
